# Temporary Botulinum Immobilization of Residuum Muscles for Facilitation of the Initial Ingrowth of Skin to the Porous *Skin and Bone Integrated Pylon* in the Technology of Direct Skeletal Attachment: Large Animal Model

**DOI:** 10.3389/fresc.2022.758238

**Published:** 2022-03-03

**Authors:** Zachary Bohart, Charles Cassidy, David Merrill, Mario Villani, Rosanna Villani, Leo Cappabianca, Mark Pitkin

**Affiliations:** ^1^Department of Orthopaedics and Physical Medicine and Rehabilitation, Tufts University School of Medicine, Boston, MA, United States; ^2^DaVinci Biomedical Research Products, Lancaster, MA, United States; ^3^Poly-Orth International, Sharon, MA, United States

**Keywords:** direct skeletal attachment, porcine model, skin immobilization, botulinum injections, osseointegration, body-implant interface

## Abstract

Enhancing the technology of bone-anchored limb prosthetics, we present a modified porcine model for developing an infection-free integration between the skin and a percutaneous bone implant. The deeply porous Skin and Bone Integrated Pylon (SBIP) presented an infection-free skin-implant interface both after implantation into the dorsum and after implantation into the residuum after below-knee amputation. However, deep ingrowth of skin into the porous cladding of the SBIP was achieved better in the dorsal procedure, while implantation to the residuum sometimes developed a stoma, probably due to the high mobility of the skin and soft tissues in the pig's thigh. Uncontrolled high skin mobility during the first week after implantation constituted a limitation for the porcine animal model, which we tried to address in the current study. As our previous studies showed that casting of the leg residuum did not sufficiently limit the skin's movement around the implant, we tested a modified protocol of the implantation, which included injection of botulinum toxin into the thigh muscles. During the course of the study, we identified proper botulinum toxin componentry, dosage, and the period after injections to achieve a maximal effect of immobilization of the muscles affecting skin movements. To verify the immobilization, we used kinetic data on the asymmetry of loading during gait with the Strideway System, Tekscan, Inc., Boston, MA, USA. We found that injections in the four muscles of the distal thigh of the left hind leg with MYOBLOC^®^ (rimabotulinumtoxinB; 5,000 units/muscle) were sufficient to provide noticeable immobilization by the fourth week after the procedure. This conclusion was made based on the analysis of the dynamics of asymmetry in vertical ground reactions on the injected (left hind) and uninvolved (right hind) legs during gait over an instrumented walkway.

## Introduction

Bone-anchored limb prostheses offer a number of advantages over socket-based prostheses (1). The technology of osseointegration relies on the integration of the residuum's bone with the titanium implant and traces its origins to the 1950s in Sweden by Dr. Per-Ingvar Brånemark (2, 3).

A problem with this technology is the still high infection rate at the interface of the skin with the implanted fixture (4–6).

Percutaneous porous devices used in bone-anchored prostheses have the potential for initial integration with the skin, as demonstrated in animal studies by various research groups (7–9). Our studies have also investigated porous implants for direct skeletal attachment, focusing on the ability of implants to invite and sustain deep skin and bone ingrowth to promote an infection-free body-device interface while maintaining the required mechanical strength. The implant we developed with such features is called the Skin and Bone Integrated Pylon (SBIP) (8, 10–13). The innovation of the SBIP lies in its patented combination of four key technological characteristics: *porosity, pore size, porosity volume fraction* (VF), and *particle size*, and a provision for the *passage for the wired neural interface*, and *protective silver coating* (10, 14).

The parameter most distinct from the prior art, which most meaningfully distinguishes the SBIP from other systems, is the *porosity VF*, which quantifies how porous the implant is (formally, *VF* is the ratio of the volume of the porous portion to the entire volume of the device).

As the SBIP implants have been designed to encourage and enable deep skin permeation, there is a critical and vulnerable period—between implantation and full permeation—that requires methodological advances. Until the surrounding skin cells remodel within all of the implant's pores, special care to minimize the skin movements around the implant is required to protect the still non-occupied pores from bacterial infiltration (15–17). Minimizing skin movements during the initial period after transdermal implantation is especially important in the studies with large animals (pigs), since the activity of the massive musculature in the residuum and above may mechanically pull out the skin around the implant.

Our previous studies with pigs (18) showed that deep and sustainable ingrowth of skin into the porous cladding of the SBIP can be achieved after implantation into the pig's dorsum. As to implantation into the residuum of the leg, the skin developed a stoma around the implant (15, 17). There is an excess of the movable skin and soft tissues in the pig thigh; simple casting did not successfully immobilize the skin while the skin seal was developing.

Since our overall intention is to establish a sustainable and safe skin seal to provide natural barriers against infection, we tested here a modified implantation protocol. The modification is the inclusion of pre-implantation injections of botulinum toxin to temporarily immobilize the muscles that affect the movement of skin in the implantation zone.

Botulinum toxins are approved by the Food and Drug Administration (FDA) for application in human patients (19) and are frequently used in patients with spasticity of the upper and lower limbs due to upper motor neuron disorders, spinal cord injuries, multiple sclerosis, strokes, brain injuries, and cerebral palsy (20, 21). Botulinum toxin inhibits the release of acetylcholine at the neuromuscular junction, reducing the contraction of the muscle (22).

Fewer reports are extant on botulinum toxin applications in pigs (23). That makes it necessary to judiciously select the type of toxin and its dosage, which may differ from those recommended for humans (24–26).

In the current pilot study with three animals, we calibrated both the dose and optimal timing for the implantation, which is when the botulinum toxin reaches its maximum effect. This paper presents the leading hypothesis, study design, and outcomes of the study.

## Study Design

The study protocol #DB-633, “Effect of botulinum neurotoxin serotypes A or B injections into thigh musculature of a swine,” was approved by the Institutional Animal Care and Use Committee (IACUC) at DaVinci Biomedical Research Products, Inc., Lancaster, MA, USA and by the US Army Medical Research & Development Command (UASMRDC) Animal Care and Use Review Office (ACURO) on July 16, 2020, with further approval of Amendment 1 on March 25, 2021.

The purpose of this botulinum toxin study was to determine the period when the injection's immobilization effect was the greatest on the pig leg muscles. The contraction of these muscles can compromise the initial remodeling of the skin while a sustainable seal is developing after implantation of the transdermal implant into the leg's residuum.

The best timing for the implantation is when the immobilization effect is strongest. The asymmetry of loading between the uninvolved hind leg and the hind leg with injected botulinum toxin can be used to detect the maximum immobilization.

**Figure 1:**
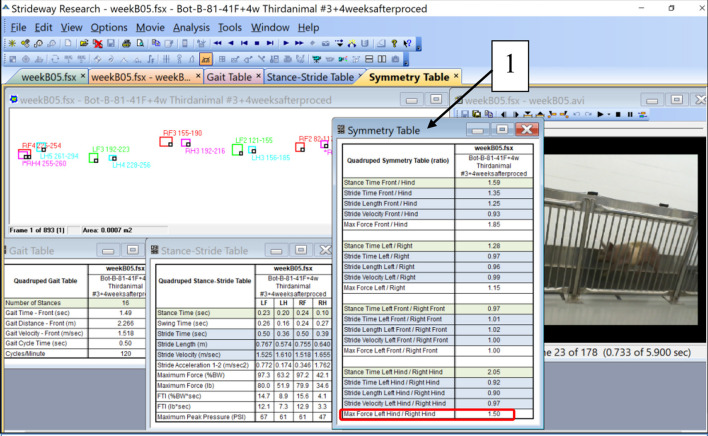
Illustration of the parameters of quadruped gait calculated by the Strideway software including a Symmetry Table (1). A ratio “Max Force Left Hind/Right Hind” at the bottom of the Symmetry Table (encircled with red) was taken as a parameter called “Asymmetry Index (AI)” (see Table 2 and the graph in Figure 3).

The intensity of immobilization was quantified by an Asymmetry Index (AI) calculated from quadruped gait analysis data obtained with the Strideway System, Tekscan, Inc. Boston, MA, USA. A standard set of data is illustrated in Figure 1. For each of the gait trials, the Strideway software, among other parameters of gait, generates a Symmetry Table as the ratios of the magnitudes of the various parameters for left and right legs. Figure 1 depicts a Symmetry Table associated with one of the five gait trials (B05) conducted with animal #3 in 4 weeks after botulinum toxin injection. The ratio “Max Force Left Hind/Rights Hind” (encircled in red square) is a parameter we called AI. We have selected this parameter for characterization of the inhibiting effect of botulinum injection on the activity of the leg muscles. An ideal magnitude of AI in sound gait, when the load on the right and left legs is equal, is 1.00.

Reports in human applications of botulinum toxin injections indicate that the mean time to peak effect is ranging from 2 weeks to 3.7 (SD ¼ 2.4) weeks, and that treatment effects declined at a mean of 9.3 (SD ¼ 4.0) weeks (27, 28).

We hypothesized that within this interval, neuro control over the muscle-coordinated activity during the gait cycle will change the magnitudes of the loading of the injected leg, as detected in the increase in the IA.

Thus, the purpose of this botulinum toxin study was to confirm this hypothesis or to make the necessary modifications in the type of botulinum toxin or its dosage.

## Methods

### Procedures

We injected botulinum toxin A (Xeomin^®^), Merz Pharma GmbH & Co., Dessau, Germany, an Incobotulinum product, equivalent to Botox^®^ and Dysport^®^ (29), and compared its effect with Botulinum toxin type B (MYOBLOC Elan Pharmaceuticals, Inc., San Francisco, CA, USA), which showed better desired effect in pigs compared to toxin A in pig masseter muscles (23). The injections were dosed at 8 units/kg, similar to human pediatrics and equivalent to the maximum allowed dose by the FDA in children to the lower limb (19).

Injections were performed using ultrasound guidance into the rectus femoris, vastus lateralis, vastus intermedius, and vastus medialis of the pigs in order to increase adherence of the below-knee prosthesis (see Table 1).

**Table 1:** Injected muscles, toxin type and dosage.

**Injected muscles**	**Toxin type and dosage**		
	**IncobotulinumA**	**RimabotulinumtoxinB (Myobloc^®^)**	
	**(Xeominò)**		
	***Animal 1 No. 1090***	***Animal 12 No. 1143F***	***Animal 3 No. 81-141F***
Rectus femoris	2.0 mL (100 units)	4.5 mL (7,500 units)	2.5 mL (5,000 units)
Vastus lateralis	2.0 mL (100 units)	4.5 mL (7,500 units)	2.5 mL (5,000 units)
Vastus intermedius	4.0 mL (200 units)	4.5 mL (7,500 units)	2.5 mL (5,000 units)
Vastus medialis	2.0 mL (100 units)	4.5 mL (7,500 units)	2.5 mL (5,000 units)
Gluteus maximus	2.0 mL (100 units)	N/A	
Total units injected	12.0 mL (600 units)	18 mL (30,000 units)	10 mL (20,000 units)

Application of the botulinum toxin treatment included injections into the distal musculature of the hind limb of the pig; daily monitoring during first 2 weeks and weekly monitoring of behavior and locomotor activity of the animal; gait analysis of the pre-procedure and following 2, 4, 6, 8, 10, and 12 weeks after the injection.

**Figure 2:**
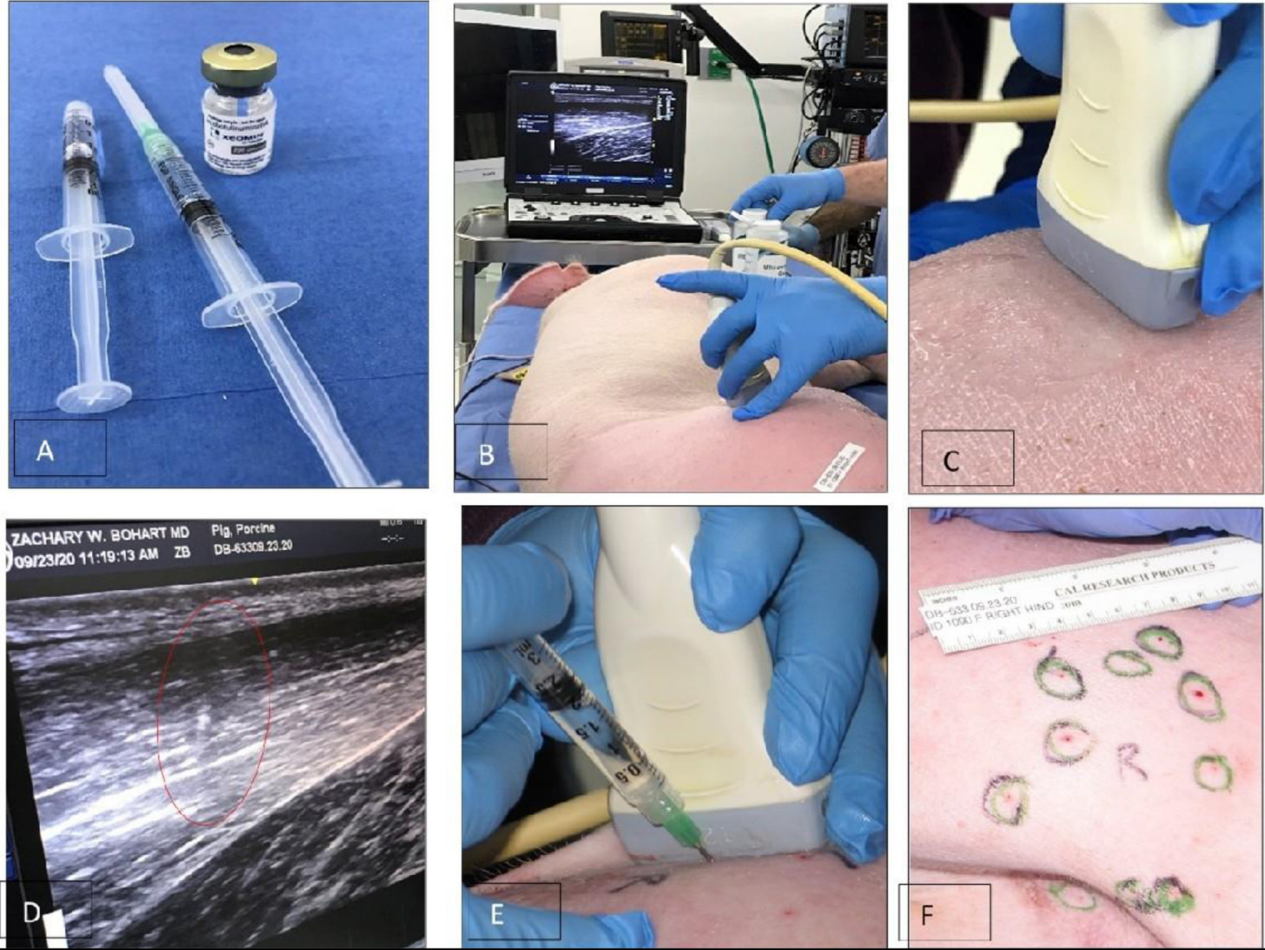
A study on immobilizing skin and muscles before the osseointegration procedure for a better integration of tissues at the skin-implant (SBIP) interface. **(A)** Xeomin^®^, an incobotulinum toxin A product equivalent to Botox^®^ and Dysport^®^. **(B)** GE Ultrasound laptop machine for guidance of injections. **(C,E)** Finding a spot for injection with visual confirmation **(D)**. **(F)** Schematics of the injection spots in the study with the Animal 1-No. 1090, 9-23-20.

Figures 2A–F illustrate the procedure of the Botulinum study in Animal 1 No. 1090. Xeomin^®^ (Figure 2A), GE Ultrasound laptop machine for guidance of injections (Figure 2B). Finding a spot for injection by moving the transducer with visual confirmation on the screen of the GE Ultrasound machine (Figures 2C–E). Schematics of the injection spots in the study, 9-23-20 (Figure 2F).

### Outcomes

#### Animal 1 No. 1090

Application of incobotulinumtoxinA was performed between September and December of 2020 by injection of botulinum toxin A to the distal thigh musculature of the right leg.

##### Outcomes

The Xeomin^®^ was ineffective. There was no muscle weakening and therefore, no effect on animal gait. For the duration of 3 months post-injection and 3 months wash-out period, the animal was normal. The animal's gait was normal throughout, with symmetrical kinematic and kinetic data compared between the involved and uninvolved legs.

The conclusion was made based on results of the consecutive gait analysis that Botulinum toxin A does not provide sufficient immobilization of the leg muscles and that a new injection with Botulinum toxin B was suggested with a modified dosage.

#### Animal 2 No. 1143F

Since the previous injections of incobotulinumtoxinA proved ineffective for blocking muscular contraction, the second cycle of application of botulinum toxin B, a different serotype of botulinum toxin treatment **[**MYOBLOC^®^ (rimabotulinumtoxinB)], was performed on January 23, 2021.

Ultrasound-guided muscular injections were entered into the distal thigh of the right hind leg, with MYOBLOC^®^ (rimabotulinumtoxinB; 5,000 units/1 ml) diluted from 1 to 3 ml using injectable saline. Four (4) muscles were each injected with 7,500 units/muscle.

The animal recovered well from the injection procedure. The animal was observed 2x daily. Observations of animal and injection sites were normal. On day 4, during AM checks, the limb appeared normal. The animal was found lying down and not eating. The animal was unable to stand and was non-responsive. After a consult with Attending Veterinarian, it was determined that there was toxicity. Animal was referred for unscheduled euthanasia. The animal was euthanized the same day.

##### Necropsy Notes

Temperature: 101.9F; heart rate: 120; respiratory rate: 20; capillary refill time: >4 s. Animal was unable to stand, lethargic. Injection sites were normal. The animal was found laterally recumbent, was paretic in the hind and front end and was slightly cyanotic.

#### Animal 3 No. 81-141F

The third animal received a smaller dosage of rimabotulinumtoxinB than Animal 2, recovered, and was tested with the Strideway gait analysis system.

Application of Botulinum treatment was performed on May 19, 2021.

UV-guided muscular injections were performed to the distal thigh of the left hind leg with MYOBLOC^®^ (rimabotulinumtoxinB; 5,000 units/1 ml) diluted from 1 to 0.5 ml using injectable saline. Four (4) muscles were each injected with 5,000 units/muscle.

Animal No. 81-141F had an uneventful recovery.

Animal was observed 2x daily. The injection sites were normal throughout the survival period. On day 6, post- injections the animal started to become paretic. This paresis lasted 6 days during which the animal was tube fed and intermittently placed in a Panepinto sling. The animal made a full recovery and was able to complete all the gait analyses.

Weekly monitoring of behavior and locomotor activity of the animal demonstrated recovery from the injection and return to regular ambulation with the greatest asymmetry in kinematic and kinetic data at week 4 after injection procedure.

**Figure 3:**
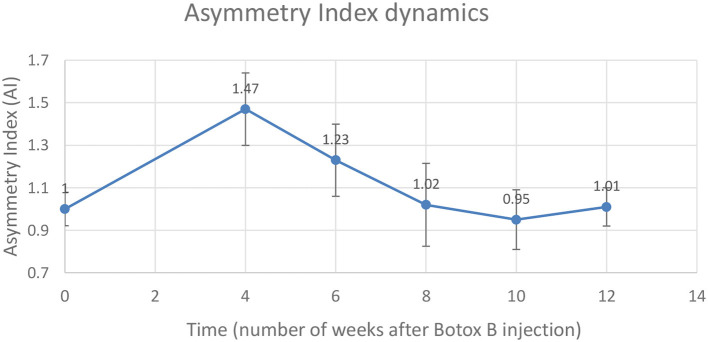
Changes in the Asymmetry Index (AI) as a ratio of maximal vertical ground reaction on the injected left leg (LH) to the uninvolved right leg (RH).

Gait analysis was performed six times: 2 days pre-procedure as a baseline, and 4, 6, 8, 10, and 12 weeks after the injection procedure. The dynamics of the AI is shown in Table 2 and is illustrated in the chart (Figure 3). A distinct increase of AI occurred at week 4 after the injection. By weeks 8–12, the AI was recovered to the initial symmetry in loading of both hind legs.

**Table 2:** Asymmetry Index dynamics over time after injection.

**Animal #3**		
**Time (weeks) after injection**	**Asymmetry index (AI)**	
	**Mean**	**STDEV**
0	1.00	0.08
4	1.43	0.21
6	1.23	0.58
8	1.02	0.19
10	1.03	0.28
12	1.01	0.09

A mean number of stance cycles was 15 (SD 1) and a mean of the walking distance was 2.17 (SD 0.10) m. The maximal AI at week 4 indicated that the loading on the injected leg by that time exceeded the loading on the uninvolved leg by 47 ± 17%. Within this interval, the neurocontrol over the muscle-coordinated activity during gait cycle was affected by the Botulinum toxin, which did not allow the leg to be lifted as quickly as the contralateral leg, which resulted in the higher magnitude of normal ground reaction.

## Discussion

We anticipated that by immobilizing the distal thigh muscles ~4 weeks before the transdermal implantation, the initial ingrowth of skin into the porous cladding will progress without being torn off by muscular movement. By that, more favorable conditions are anticipated for the creation of the skin seal at the implant-skin interface as a natural barrier against infection.

We did not consider differences among tested animals (e.g., in terms of the body morphology and sex) due to their small number, which constitutes a limitation of this pilot study.

We will investigate the benefits of the pre-implantation Botulinum injections in our further studies in bone-anchored prosthetics with this modified porcine model. The model with pre-implantation botulinum toxin injections may have higher translational value than the regular one, considering existing FDA-approved Botulinum applications in humans.

## Conclusions

1. Injections with incobotulinumtoxin^A^ (Xeomin^®^) were ineffective at inducing any form of muscle weakness with effect to gait.
2. MYOBLOC^®^ (rimabotulinumtoxinB) injections proved toxic with the first dosage applied. A range finding study was recommended to identify the optimal dose to induce muscle weakness.
3. A smaller dosage of MYOBLOC^®^ (rimabotulinumtoxinB) showed safe outcomes of the injection and demonstrated the effect expected—asymmetry (47 ± 17%) in loading between affected and non-affected limbs 4 weeks after the injection (Figure 2) compared to baseline recording (Figure 1). Further observations showed recovery of the symmetry in gait parameters: as 23 ± 21% in 6 weeks, 2 ± 21% in 8 weeks, and 3 ± 21% 10 weeks after the injection procedure (see Table 2, Figure 2).
4. Limitations of the study include a small number of animals and the pilot selection of the dosage is found effective. For addressing these limitations further studies are suggested.

## Data Availability Statement

The original contributions presented in the study are included in the article/supplementary material, further inquiries can be directed to the corresponding author.

## Ethics Statement

The animal study was reviewed and approved by IACUC DaVinci Biomedical research Products and by ACURO UASMRDC.

## Author Contributions

ZB participated in the study design and injection methodology. CC participated in injection procedures and the translational value of the modified protocol. DM, RV, MV, and LC provided animal husbandry, enrichment, and veterinary care. MP contributed to the study design and gait analysis. All authors contributed to the article and approved the submitted version.

## Funding

This study was supported by the U.S. Department of Defense grant W81XWH-16-1-0791 and by Eunice Kennedy Shriver National Institute of Child Health and Human Development, the NIH grants HD057492 and HD090768.

## Conflict of Interest

DM, MV, RV, and LC were employed by company DaVinci Biomedical Research Products. MP was employed by Poly-Orth International. The remaining authors declare that the research was conducted in the absence of any commercial or financial relationships that could be construed as a potential conflict of interest.

## Publisher's Note

All claims expressed in this article are solely those of the authors and do not necessarily represent those of their affiliated organizations, or those of the publisher, the editors and the reviewers. Any product that may be evaluated in this article, or claim that may be made by its manufacturer, is not guaranteed or endorsed by the publisher.
